# Escape of HIV-1-Infected Dendritic Cells from TRAIL-Mediated NK Cell Cytotoxicity during NK-DC Cross-Talk—A Pivotal Role of HMGB1

**DOI:** 10.1371/journal.ppat.1000862

**Published:** 2010-04-15

**Authors:** Marie-Thérèse Melki, Héla Saïdi, Alexandre Dufour, Jean-Christophe Olivo-Marin, Marie-Lise Gougeon

**Affiliations:** 1 Institut Pasteur, Antiviral Immunity, Biotherapy and Vaccine Unit, Paris, France; 2 Institut Pasteur, Quantitative Image Analysis Unit, CNRS URA 2582, Paris, France; University of Pennsylvania School of Medicine, United States of America

## Abstract

Early stages of Human Immunodeficiency Virus-1 (HIV-1) infection are associated with local recruitment and activation of important effectors of innate immunity, i.e. natural killer (NK) cells and dendritic cells (DCs). Immature DCs (iDCs) capture HIV-1 through specific receptors and can disseminate the infection to lymphoid tissues following their migration, which is associated to a maturation process. This process is dependent on NK cells, whose role is to keep in check the quality and the quantity of DCs undergoing maturation. If DC maturation is inappropriate, NK cells will kill them (“editing process”) at sites of tissue inflammation, thus optimizing the adaptive immunity. In the context of a viral infection, NK-dependent killing of infected-DCs is a crucial event required for early elimination of infected target cells. Here, we report that NK-mediated editing of iDCs is impaired if DCs are infected with HIV-1. We first addressed the question of the mechanisms involved in iDC editing, and we show that cognate NK-iDC interaction triggers apoptosis via the TNF-related apoptosis-inducing ligand (TRAIL)-Death Receptor 4 (DR4) pathway and not via the perforin pathway. Nevertheless, once infected with HIV-1, DC_HIV_ become resistant to NK-induced TRAIL-mediated apoptosis. This resistance occurs despite normal amounts of TRAIL released by NK cells and comparable DR4 expression on DC_HIV_. The escape of DC_HIV_ from NK killing is due to the upregulation of two anti-apoptotic molecules, the cellular-Flice like inhibitory protein (c-FLIP) and the cellular inhibitor of apoptosis 2 (c-IAP2), induced by NK-DC_HIV_ cognate interaction. High-mobility group box 1 (HMGB1), an alarmin and a key mediator of NK-DC cross-talk, was found to play a pivotal role in NK-dependent upregulation of c-FLIP and c-IAP2 in DC_HIV_. Finally, we demonstrate that restoration of DC_HIV_ susceptibility to NK-induced TRAIL killing can be obtained either by silencing c-FLIP and c-IAP2 by specific siRNA, or by inhibiting HMGB1 with blocking antibodies or glycyrrhizin, arguing for a key role of HMGB1 in TRAIL resistance and DC_HIV_ survival. These findings provide evidence for a new strategy developed by HIV to escape immune attack, they challenge the question of the involvement of HMGB1 in the establishment of viral reservoirs in DCs, and they identify potential therapeutic targets to eliminate infected DCs.

## Author Summary

Dendritic cells (DCs), the professional antigen presenting cells, are critical for host immunity by inducing specific immune responses against a broad variety of pathogens. Human Immunodeficiency Virus-1 (HIV-1) has evolved ways to exploit DCs, thereby facilitating viral dissemination and allowing evasion of antiviral immunity. In particular, infected DCs may function as cellular reservoirs for HIV-1, thus contributing to viral persistence in lymphoid tissues. The mechanisms involved in the constitution of HIV reservoirs in DCs are poorly understood. In this study, we reveal that DCs infected with HIV-1 (DC_HIV_) become resistant to killing by natural killer (NK) cells, early effectors of innate immunity involved in the destruction of virus infected cells or cancer cells. This protection of DC_HIV_ from NK cytotoxicity is induced through a cross-talk between NK cells and DC_HIV_, which induces the upregulation in DC_HIV_ of two inhibitors of cell death, i.e. cellular-Flice like inhibitory protein (c-FLIP) and cellular inhibitor of apoptosis 2 (c-IAP2). The molecule responsible for the induction of these inhibitors is High-mobility group box 1 (HMGB1), an alarmin involved in the functional maturation of DCs. Blocking HMGB1 restores DC_HIV_ susceptibility to NK cell killing, arguing for a key role of HMGB1 in the persistence of DC_HIV_. These findings provide evidence of the crucial role of NK-DC cross-talk in promoting viral persistence, and they identify potential therapeutic targets to eliminate infected DCs.

## Introduction

Dendritic cells (DCs) are crucial for the generation and the regulation of adaptive immunity. Immature DCs (iDCs) sample the environment via pattern recognition receptors such as Toll-like receptors (TLRs), and they undergo a maturation process characterized by increased expression of HLA class I proteins and surface molecules (CCR7, CD80, CD86, HLA class II), and secretion of proinflammatory cytokines and chemokines. Resulting mature DCs migrate to secondary lymphoid tissues, where they prime an antigen-specific T cell response [1]. Recently, the fate of DCs has been found to be extremely dependent on NK cells [2]. After being recruited into inflamed tissues, NK cells can interact with iDCs, resulting in their activation that, in turn, induce DC maturation or killing, depending on their respective density [3] [4] [5]. Thus, one of the major roles of NK cells is to keep in check the quality and the quantity of DCs undergoing maturation [6]. DC maturation is dependent on TNF-α produced by activated NK cells [4], but it also involves the alarmin high-mobility group box 1 protein (HMGB1) released at the synaptic cleft in response to IL-18 produced by DCs [7]. HMGB1 is a highly mobile nuclear protein that functions to stabilize nucleosome formation, and acts as a transcription-factor-like protein that regulates the expression of several genes [8]. HMGB1 is either secreted actively from inflammatory cells (activated macrophages and NK cells in response to inflammatory stimuli), or passively released from necrotic cells to signal tissue injury. It triggers a cascade of inflammatory responses through its binding to receptor for advanced glycation end products (RAGE), TLR2, or TLR4 expressed on monocytes, macrophages and NK cells [9]–[11]. As an alarmin, HMGB1 has mobilizing and activating effects for host defense [12], it facilitates the trafficking of inflammatory leukocytes, and it is critical for DCs to mature, reach the lymph nodes and promote polarization of antigen-specific T cells towards a Th1 phenotype [13]–[16].

The cognate NK-DC interaction in inflamed tissues can also result in acquisition of NK cytotoxicity against iDCs, which may represent a mechanism of DC selection required for the control of downstream adaptive immune response [6]. This editing process is dependent upon the engagement of NKp30 [17] [18] and DNAM-1 [19] by ligands expressed on iDC, and the down-regulation on iDC of HLA-E, the ligand for CD94/NKG2A inhibitory receptor on NK cells [20]. While NK-dependent killing of allogeneic DCs in a murine model of skin graft rejection was reported to involve the perforin pathway [21], the cytotoxic pathway involved in the killing of syngeneic iDCs has not been identified yet.

In the context of a viral infection, NK-dependent killing of infected-DCs is a crucial event required for early elimination of infected target cells. Indeed, in murine CMV infection, infected DCs are capable of activating NK cell cytotoxicity *in vitro* and also able to enhance NK-cell dependent virus clearance *in vivo* [22]. HIV has evolved ways to exploit DCs, allowing evasion of antiviral immunity. Recent reports suggest that NK-DC interactions are altered in HIV-1-infection. A defect in NK cell lysis of immature monocyte-derived DCs generated from HIV-1-infected individuals has been reported [23]. In viremic patients, *in vitro* interactions between a CD56^neg^/CD16^pos^ subset of NK cells and autologous DCs were found markedly impaired, evidenced by abnormalities in the process of reciprocal NK-DC activation and maturation, as well as a defect in NK-cell elimination of iDCs [24]. However, the mechanisms involved in this escape from NK cytotoxicity have not yet been elucidated.

In this study, we first identified the molecular pathway implicated in the editing process of non-infected iDCs by NK cells and found that it did not involve the perforin pathway but rather the TRAIL/DR4-dependent death receptor pathway. We then addressed the question of the mechanisms involved in the resistance of HIV-1-infected DCs (DC_HIV_) to NK-dependent killing, and found that it was linked to the dramatic upregulation of c-IAP2 and c-FLIP in DC_HIV_, induced by the cognate interaction with NK cells, and leading to the resistance of DCs to TRAIL-dependent apoptosis. Furthermore, HMGB1, a key mediator of NK-DC cross-talk [7], [16], was found to play a pivotal role in the process by upregulating c-IAP2 and c-FLIP in infected DCs. At present, these data provide evidence for a new strategy developed by HIV-1 to escape immune attack through the induction in DCs of a potent anti-apoptotic mechanism leading to their escape from innate cytotoxicity. They also challenge the question of the involvement of HMGB1 in the establishment of viral reservoirs in DCs, and the possible destruction of these reservoirs by c-IAP2 and c-FLIP antagonists.

## Results

### Cytotoxic activity of NK cells on autologous primary iDCs

**Figure 1:**
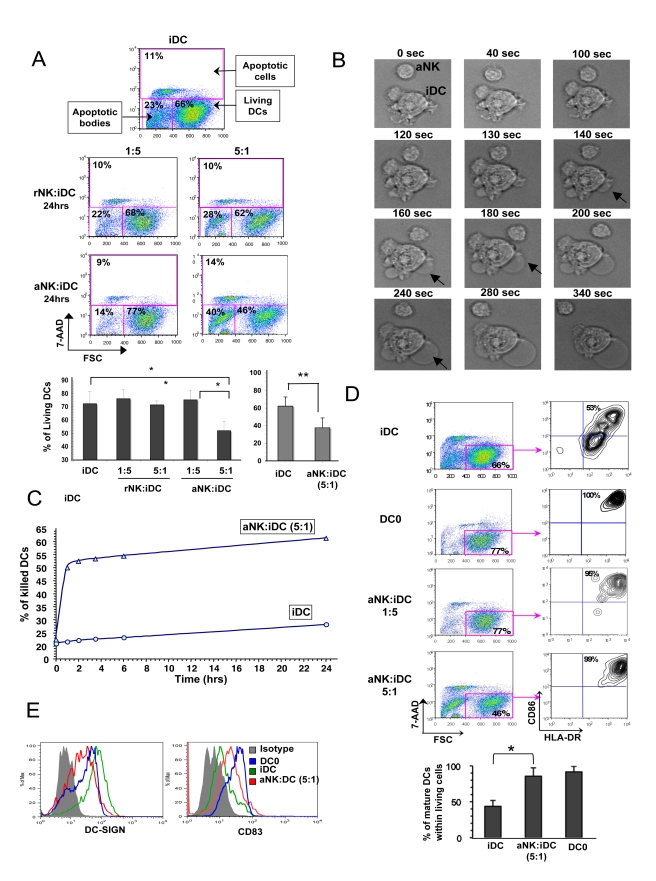
aNK cells induce apoptosis of primary immature DCs. (**A**) iDCs, generated from purified CD14^+^ monocytes in the presence of IL-4 and GM-CSF, were co-cultured during 24 h with rNK or aNK at two different NK∶DC ratios (1∶5 and 5∶1). DC survival was determined by flow cytometry with the 7-AAD assay. Surviving DCs were identified as CD56^neg^ 7-AAD^neg^ FSC^high^ cells. Histograms (left side) show the mean ± sd of three independent experiments * p<0.05, and histograms (right side) show the mean ± sd of experiments performed with cells from 14 healthy donors ** p<0.00001). (**B**) Kinetics of NK-mediated DC apoptosis observed by live video microscopy. The arrows point out the appearance of blebs in iDCs. Pictures from one representative out of three experiments are shown. (**C**) Kinetics of iDC apoptosis, measured as described in (A), cultured alone or in the presence of aNK cells. Data representative from one out of three independent experiments are shown. (**D**) Analysis of the maturation stage of surviving DCs, after 24 h of culture in medium (iDC) or in the presence of aNK cells (ratio 1∶5 and 5∶1), or induced to mature with LPS (DC0). Mature cells are identified through the co-detection of surface HLA-DR and CD86. The proportions of double positive cells are indicated. Histograms show the mean ± sd of three independent experiments * p<0.05. (**E**) Surface expression of DC-SIGN and CD83 in surviving DCs cultured as described in (D). Data are representative of one out of three independent experiments.

To investigate the impact of NK cells on the fate of DCs, iDCs were generated from monocytes sorted from healthy donors and cocultured with autologous purified NK cells (all sorted NK cells expressed CD56). NK cells were kept either unstimulated (rNK) or were activated (aNK) with a combination of PHA and IL-2. The fate of iDCs in 24h NK-DC cocultures was studied by flow cytometry after exclusion from the analysis of CD56^+^ cells (NK cells). 24 h coculture of aNK cells with autologous iDCs induced either the survival or apoptosis of iDCs, depending on aNK∶DC ratio, consistent with previous reports [4]. Indeed, at low aNK-DC ratios (1∶5), this interaction induced DC maturation and cytokine production, as previously reported [4], [16], while higher NK∶DC ratios (5∶1) induced DC killing by autologous NK cells [4] (**Fig. 1A**). FSC/7-AAD dot plots distinguish living (7-AAD^neg^ FSC^high^) from apoptotic (7-AAD^pos^ FSC^low^) DCs and apoptotic bodies (7-AAD^neg^ FSC^low^) [25]. Reduced DCs survival combined with the accumulation of apoptotic bodies was detected at high aNK∶DC ratio (5∶1), while normal iDC survival and no accumulation of apoptotic bodies was observed at aNK∶DC ratio of 1∶5, as compared with iDCs cultured alone (**Fig. 1A**). Under the same conditions, rNK cells had no impact on the fate of iDCs and did not kill them (**Fig. 1A**). In order to confirm that apoptotic cells and bodies were generated from iDCs, two additional approaches were used. In the first one, the level of apoptosis in gated CD56^+^ aNK cells was compared when cultured alone or in the presence of iDCs. **Figure S1** (panel A) shows that there was no induction of apoptosis in aNK cells after their cross-talk with iDCs (ratio 5∶1), and no increase of apoptotic bodies either (not shown). In the second approach, aNK cells were stained with CFSE prior to their coculture with iDCs, and apoptosis was analyzed in Carboxyfluorescein succinimidyl ester (CFSE)^neg^ cells, e.g. DCs. **Figure S1** (panel B) confirms that, at 5∶1 ratio, aNK cells kill iDCs, as shown by the increased percentages of apoptotic cells (7-AAD^pos^ FSC^low^) and apoptotic bodies (7-AAD^neg^ FSC^low^) in CFSE^neg^ cells in aNK∶DC cocultures compared to iDCs cultured alone. Live video microscopy pictures in **Fig. 1B** show the kinetics of events following the rapid contact of one NK cell with one DC, leading to an NK cell kiss of death, inducing immediate plasma membrane blebbling in the DC, a classical feature of apoptotic death (see the video http://www.bioimageanalysis.org/plospathogens2010_suppldata). Death triggering of iDCs by aNK cells is a very rapid process, detected a few seconds after cell contact at the single cell level (**Fig. 1B**), and reaching a plateau after 1 hour in bulk cultures, as measured by 7-AAD staining in 24 h kinetics experiments (**Fig. 1C**). Since the editing process of iDCs by NK cells is supposed to keep in check the quality of DCs undergoing maturation [6], we assumed that DCs surviving the interaction with aNK cells were mature. **Fig. 1D** shows that survival of DCs in aNK∶iDC cocultures was associated with their maturation, as demonstrated by the coexpression of the maturation markers CD86 and HLA-DR [16] in all DCs. Similar characteristics were observed in control mature DC0, induced by 48 h stimulation with LPS (**Fig. 1D**). The expression of two other maturation markers, DC-SIGN and CD83 was investigated (**Fig. 1E**). Surviving iDCs in aNK∶DC cocultures showed increased expression of CD83 and decreased expression of DC-SIGN as compared to DC cultures alone, confirming their mature stage.

### Apoptosis of iDCs induced by aNK cells is not perforin - dependent

**Figure 2:**
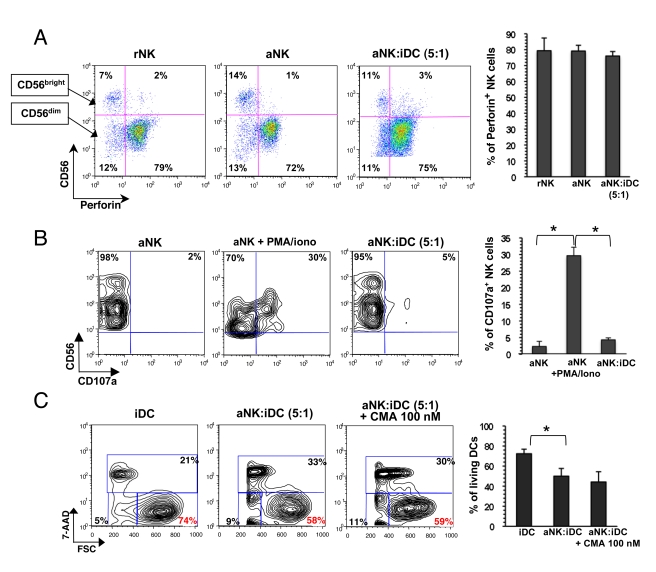
aNK-mediated killing of iDCs is independent of the perforin pathway. (**A**) Analysis of intracellular perforin expression in sorted primary rNK cells, or aNK cells cultured either alone or in the presence of iDCs for 24 h. Dot plots represent perforin expression in CD56^dim^ and CD56^bright^ NK cells. Histograms show the mean ± sd of three independent experiments (perforin expression was similar under the three conditions). (**B**) Surface CD107a expression in either aNK cells, or aNK cells stimulated by PMA+ionomycin or cocultured with iDCs for 24 h. The proportions of both CD56^+^CD107a^+^ and CD56^+^CD107a^−^ cells are indicated. Histograms show the mean ± sd of three independent experiments * p<0.05. (**C**) iDCs were cultured for 24 h either alone or with aNK cells, in the absence or presence of concanamycin A (CMA), an inhibitor of perforin-based cytotoxic activity. Surviving DCs, identified as CD56^neg^ 7-AAD^neg^ FSC^high^ cells, were quantified and the percentages indicated in red. Histograms show the mean ± sd of three independent experiments * p<0.05.

Classically, NK cells kill their targets through the perforin/granzyme pathway. To determine its possible involvement in NK-dependent killing of iDCs, intracellular perforin staining was compared in rNK, aNK, and aNK-iDC cocultures. Perforin was found mostly expressed in CD56^dim^ NK cells and activation of NK cells did not induce marked changes in perforin expression. Following coculture with iDCs, aNK cells did not express more perforin (**Fig. 2A**), suggesting that this cytotoxic pathway was not used to kill iDCs. This was confirmed by the lack of CD107a externalization when aNK cells were cocultured with iDCs, while NK stimulation with PMA/ionomycin induced CD107a expression (**Fig. 2B**). These observations were made under conditions whereby aNK cells were able to kill iDCs, from 1 to 24 hours of coculture. Furthermore, the addition of an inhibitor of perforin-mediated lysis, Concanamycin A (CMA), at concentrations ranging from 50 to 500 nM, was unable to block NK-dependent killing of iDCs (**Fig. 2C**). Altogether, these data are not in favor of the involvement of the perforin pathway in the NK-dependent editing process of iDCs.

### NK cell-dependent editing of iDCs involves the TRAIL/DR4 pathway

**Figure 3:**
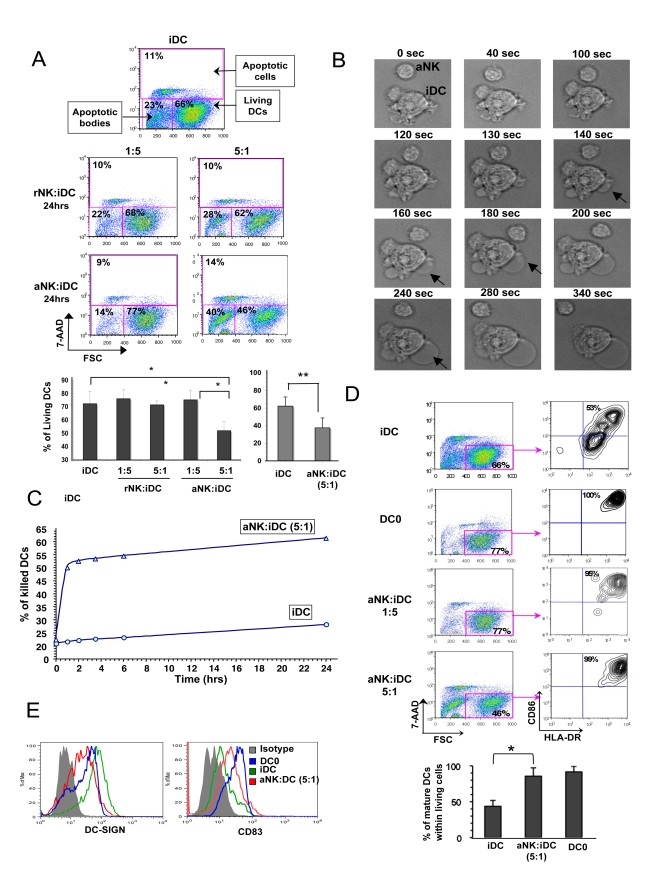
NK cell-dependent editing of iDCs involves the TRAIL/DR4 pathway. (**A**) Membrane TRAIL (mTRAIL) expression by aNK cells was analyzed after surface dual staining with anti-CD56 and anti-mTRAIL specific antibodies. The percentage of mTRAIL-expressing aNK cells among either total CD56^+^, or CD56^bright^ or CD56^dim^ subsets is shown. Data represent the mean ± sd of three independent experiments. * p<0.05. (**B**) Soluble TRAIL (sTRAIL) was quantified by ELISA in 24h cell-free culture supernatants from iDCs (10^6^/ml), rNK cells (10^6^/ml), aNK cells (10^6^/ml), or aNK-iDCs cocultures at 1∶5 (2×10^5^ NK∶10^6^ DCs), 1∶1 (2×10^5^ NK: 2×10^5^ DCs) and 5∶1 (10^6^ NK: 2×10^5^ DCs) ratios. The dotted line shows the limit of sTRAIL detection. Data represent the mean ± sd of three independent experiments. * p<0.05. (**C**) iDCs were cultured for 24 hours with increasing concentrations (1–500 ng/ml) of recombinant human soluble TRAIL (rhs-TRAIL). Cell death was quantified with the 7-AAD assay and surface expression of DR4 on DCs was analyzed by flow cytometry. Data represent the mean ± sd of three independent experiments. (**D**) 24 h kinetics of DR4 expression at the surface of DCs that were cultured alone or with aNK at aNK∶iDC ratio of 5∶1. Grey and orange histograms indicate the proportions of dead and living DR4^+^DCs respectively, among total DR4^+^DCs. Inserted dot plots show that the great majority of 7-AAD^+^ DCs are DR4^+^ after 24 h of coculture with aNK cells. Data from one representative out of three experiments are shown. (**E**) Membrane expression of DR4, DR5 and Fas on iDCs cultured either alone or with aNK cells for 24 h was detected by flow cytometry. aNK cells were excluded from the analysis by gating on CD56^neg^ cells. Dot plots from one representative out of three experiments are shown. (**F**) The impact of neutralizing anti-TRAIL antibodies on DR4 expression and killing of iDCs was tested in 24 h aNK-iDC cocultures. The percentage of 7-AAD/DR4 single and double positive populations are indicated in the quadrants. Dot plots from one out of three experiments are shown. (**G**) The impact of either anti-DR4 or anti-Fas antibodies on aNK-mediated killing of iDCs in a 24 h coculture is shown. Early and late apoptotic DCs (gated as CD56^neg^) are identified by 7-AAD^low^ and 7-AAD^high^ staining, as reported [25]. Dot plots are representative of one of three independent experiments.

TNF-related apoptosis-inducing ligand (TRAIL) is active in two forms, either expressed at the cell membrane (mTRAIL), or as a soluble form secreted in the cell environment (sTRAIL). mTRAIL expression on CD56^+^ aNK cells was found restricted to CD56^bright^ NK cells (**Fig. 3A**). However, following their contact with iDCs, both CD56^bright^ and CD56^dim^ NK cells expressed mTRAIL suggesting that killing of iDCs was not restricted to CD56^bright^ cells (data not shown). sTRAIL was mainly secreted by aNK cells, including in cocultures of aNK∶iDC, under conditions whereby iDCs were killed by aNK cells (NK∶DC ratio of 5∶1) (**Fig. 3B**). iDCs did not express mTRAIL, whether cultured alone or in the presence of NK cells (data not shown), and accordingly they did not release sTRAIL in culture supernatant (**Fig. 3B**). iDCs were susceptible to rhsTRAIL, which in turn induced DR4 expression on these cells (**Fig. 3C**). 24h kinetic experiments of DC-killing by aNK cells showed the progressive increase of apoptotic DCs within DR4^+^DCs, arguing for the involvement of DR4 receptor in the death of iDCs (**Fig. 3D**). DR5 expression was not induced on iDCs by aNK cells, ruling out its involvement in the apoptosis process (**Fig. 3E**), neither was Fas expressed on iDCs under the same conditions (**Fig. 3E**). The involvement of the TRAIL/DR4 pathway in aNK-dependent killing of iDCs was confirmed by the blocking effect of anti-TRAIL antibodies (1µg/ml) that resulted in a significant decrease in DR4^+^ apoptotic DCs (**Fig. 3F**). Moreover, anti-DR4 antibodies abrogated aNK-induced apoptosis of iDCs, while anti-Fas antibodies had no effect (**Fig. 3G**). Collectively, these results indicate that the pathway used by aNK cells to kill iDCs involves the death ligand TRAIL, whose release by NK cells induces DR4 receptor on iDCs, followed by their killing.

### HIV-1-infected iDCs are resistant to NK-dependent TRAIL-mediated killing

**Figure 4:**
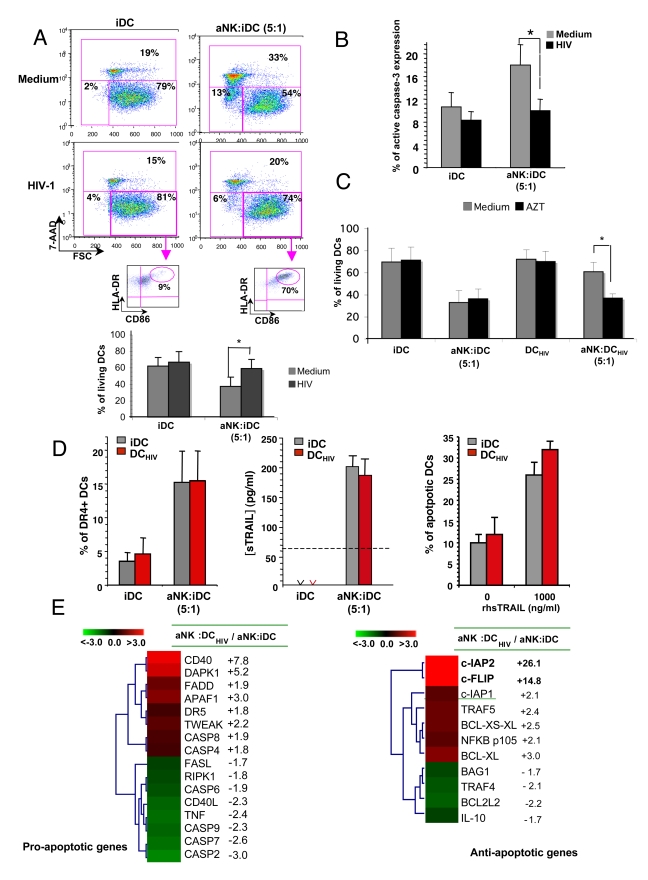
HIV-infected iDCs are resistant to NK-mediated killing. (**A**) iDCs were either uninfected or infected for 24 hours with R5-HIV-1_BaL_ (1 ng/ml of p24), and further incubated for 24 hours with aNK cells at NK∶DC ratio of 5∶1. Viability of iDCs was assessed using the 7-AAD assay. Living DCs are identified as CD56^neg^ 7-AAD^neg^ FSC^high^. Inserts: the maturation stage of DCs was determined via the dual staining of gated living DCs with anti-CD86 and -HLA-DR specific antibodies. Dot plots show one representative out of more than ten independent experiments. Histograms show the mean ± sd of experiments performed with cells from 10 healthy donors. * p = 0.0001. (**B**) Intracellular active caspase-3 detection by flow cytometry in iDC and DC_HIV_ cultured alone, or cocultured with aNK cells for 24 hours. Data represent the mean ± sd of three independent experiments. * p<0.05. (**C**) iDCs were infected for 24 hours with R5-HIV-1_BaL_ (1 ng/ml of p24) in the presence or absence of AZT 1mM, and further incubated either alone or with aNK cells at NK∶DC ratio of 1∶5. Uninfected iDCs were submitted to the same culture conditions. Viability of iDCs was assessed using the 7-AAD assay, as detailed for panel A (**D**) iDCs or DC_HIV_, cultured either alone or in the presence of aNK cells, were compared for DR4 expression on DCs analyzed by flow cytometry (left panel), sTRAIL release in culture supernatant, detected by ELISA (middle panel), or susceptibility to recombinant TRAIL-induced apoptosis, analyzed with the 7-AAD assay (right panel). The mean ± sd of three independent experiments is shown. (**E**) Comparative gene array expression between aNK∶iDC_HIV_ and aNK∶iDC was performed as described in the Material and Methods section. The expression profiles are shown for proapoptotic and antiapoptotic genes.

To address the question of the impact of HIV-1 on DC's susceptibility to NK killing, iDCs were infected with 1ng of p24/ml of R5-HIV-1_BaL_, 24 h prior to coculture with aNK cells. HIV-1-infected iDCs (DC_HIV_) were found resistant to aNK-induced cytotoxicity, the proportions of living DCs (FSC^high^ 7-AAD^low^) being unchanged after cognate interaction with aNK cells (**Fig. 4A**). It was also evidenced by the lack of caspase-3 activation in DC_HIV_ under the same culture conditions (**Fig. 4B**). Surviving DCs exhibited a mature phenotype, as shown by the co-expression of CD86 and HLA-DR on their cell surface (**Fig. 4A**). DC_HIV_ resistance to NK killing was dependent on HIV-1 replication in DCs. Indeed, adding AZT at the initiation of iDCs infection (1ng/ml of R5-HIV-1_BaL_), at the concentration of 1mM that completely inhibited HIV-1 replication in these cells (as shown by the lack of detection of p24 in culture supernatant), preserved the editing process (e.g. susceptibility of DC_HIV_ to NK killing) (**Fig. 4C**). Thus, productive HIV-1 infection of DCs was required for their acquired resistance to NK-mediated cytotoxicity (**Fig. 4C**). Resistance of DC_HIV_ to NK cell killing was independent of DR4 expression, the frequency of DR4^+^ cells being similar in infected vs. uninfected DCs (**Fig. 4D**, left panel), and the amount of sTRAIL released by aNK cells was comparable in NK∶DC vs NK∶DC_HIV_ cocultures (**Fig. 4D**, middle panel). Moreover, DC_HIV_ were found to be as susceptible as uninfected iDCs to recombinant sTRAIL (**Fig. 4D**, right panel). Thus, DC_HIV_ resistance to NK cytotoxicity was not associated with defective TRAIL release by NK cells or altered DR4 expression by DC_HIV_. In order to identify key molecules involved in the resistance of DC_HIV_ to NK killing, gene array experiments were performed and differential gene expression was compared between aNK∶DC_HIV_ vs aNK∶iDC. The expression profiles shown in **Fig. 4E** indicate a decrease in the expression of some caspase genes (Caspase-2, -6, -7 and -9), or death-ligand genes (e.g. TNF or FasL) in cocultures of aNK-iDC_HIV_,. In parallel, a dramatic upregulation of two anti-apoptotic genes, c-IAP2 (26.1 fold increase) and c-FLIP (15 fold) was detected in aNK∶DC_HIV_ as compared to aNK∶iDC cocultures (**Fig. 4E**). The expression of Bcl-2 and Mcl-1 was unchanged, whether tested in gene array experiments, or by flow cytometry in DC_HIV_ in the presence or absence of NK cells (data not shown).

### Resistance of HIV-1-infected iDCs to aNK-dependent killing is associated with increased expression of c-FLIP and c-IAP2

**Figure 5:**
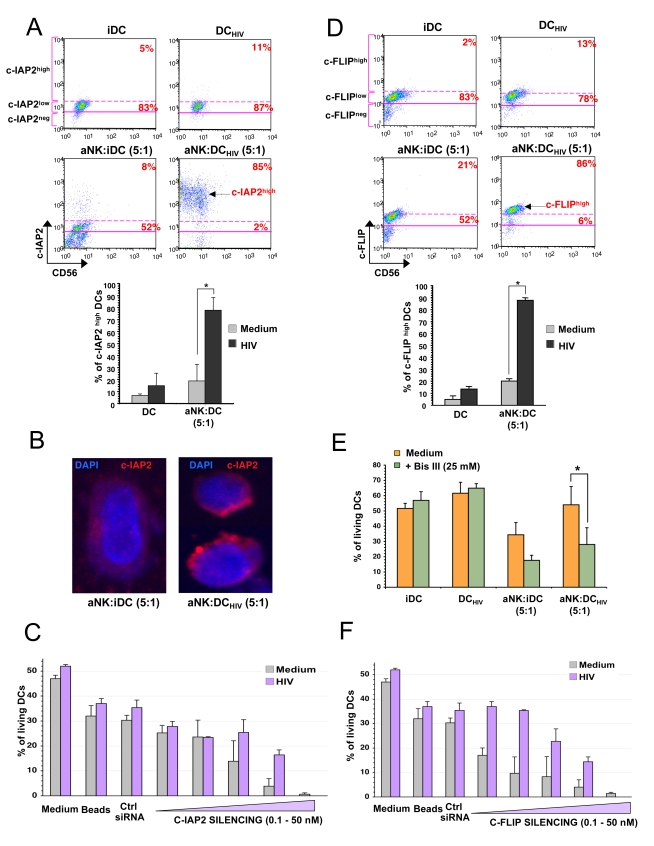
Resistance of HIV-infected iDCs to NK killing is associated with increased expression of c-IAP2 and c-FLIP. (**A**) Upper panel: analysis by flow cytometry of intracellular expression of c-IAP2 in DCs that were either uninfected or infected with R5-HIV-1_BaL_ and cultured for 24 h either alone or in the presence of aNK cells. The percentage of c-IAP2^high^ cells within DCs is indicated. Lower panel: histograms represent the mean percentage ± sd of c-IAP2^high^ DCs under indicated conditions in four independent experiments. * p = 0.02. (**B**) Confocal microscopy analysis of uninfected iDCs or DC_HIV_ costained with DAPI and c-IAP2 specific antibodies. (**C**) Uninfected DCs or DC_HIV_ were transfected with control siRNA or increasing concentrations (0.1–50 nM) of c-IAP2 specific siRNA that were coupled to beads, as described in the Material and Methods section. Uncoupled beads were used as toxicity control. Cell death was quantified with the 7-AAD assay. Mean ± sd values of three independent experiments are shown. (**D**) Upper panel: flow cytometry analysis of intracellular expression of c-FLIP in iDC that were either uninfected or HIV-infected and then cultured alone or in the presence of aNK cells for 24 h. The percentage of c-FLIP^high^ DCs is indicated. Lower panel: histograms represent the mean percentage ± sd of c-FLIP^high^ DCs from three independent experiments. * p = 0.04. (**E**) iDCs or DC_HIV_ were treated with 25 µM of Bisindolylmalmeimide III (Bis III) and then cultured alone or in the presence of aNK cells for 24 h. Survival of DCs was quantified by flow cytometry (7-AAD assay) on gated CD56^neg^ cells. The mean ± sd of three independent experiments is shown. * p<0.05. (**F**) iDCs or DC_HIV_ were transfected with control siRNA or increasing concentrations (0.1–50 nM) of c-FLIP specific siRNA coupled to beads, as described in Material and Methods section. Uncoupled beads were used as toxicity control. Cell death was quantified with the 7-AAD assay. Mean values ± sd of three independent experiments are shown.

The strong impact of aNK cells on c-IAP2 upregulation in DC_HIV_ during NK-DC cross-talk is shown in **Fig. 5A** Intracellular flow cytometry analysis shows that c-IAP2 is expressed in the majority of surviving DCs at low levels, whether infected or not by HIV-1. Cognate interaction of DC_HIV_ with aNK cells induced a strong up-regulation of c-IAP2 in the great majority of DC_HIV_ (CD56^neg^), whereas it had no effect on uninfected iDCs under the same conditions (85% vs 8% of c-IAP2^bright^ cells) (**Fig. 5A**). HIV-1 infection by itself only slightly increased c-IAP2 expression in iDCs (11% in DC_HIV_ vs 5% in iDCs of c-IAP2^bright^ cells). Thus, resistance of DC_HIV_ to NK killing is associated with the upregulation of c-IAP2 in these cells. c-IAP2 up-regulation in DC_HIV_ following their cognate interaction with aNK cells was confirmed by confocal microscopy (**Fig. 5B**). DC survival was strongly dependent upon c-IAP2 expression. Indeed, the knock-down of c-IAP2 with specific siRNA induced apoptosis of both uninfected iDCs and DC_HIV_, in a dose-dependent manner (**Fig. 5C**). The expression of another anti-apoptotic molecule was identified by microarray analysis as being upregulated in DC_HIV_ following their contact with aNK cells, i.e. c-FLIP (**Fig. 4D**). This was confirmed by intracellular flow cytometry analysis, showing that c-FLIP expression was slightly increased in DC_HIV_ vs iDCs (13% vs 5%), whereas a strong induction of c-FLIP was observed following their interaction with aNK cells (86% of c-FLIP^high^ in DC_HIV_ vs 21% in iDC) (**Fig. 5D**). The key role of c-FLIP on DC resistance to NK killing was confirmed using a specific inhibitor Bisindolylmalmeimide III (Bis III). Blocking c-FLIP activity with Bis III added at 25µM in NK-DC_HIV_ cocultures significantly restored the susceptibility of HIV-1-infected DCs to NK cytotoxicity, whereas it preserved the survival of iDC or DC_HIV_ cultured alone (**Fig. 5E**). The involvement of c-FLIP in DC survival was confirmed with specific siRNAs. Indeed, the knock-out of c-FLIP induced apoptosis of iDCs, in a dose-dependent manner (**Fig. 5F**). Strikingly, higher siRNA concentrations were needed to induce apoptosis in DC_HIV_ as compared to iDCs (**Fig. 5F**). Overall these silencing experiments show the essential role of c-FLIP and c-IAP2 on the survival of primary DCs, which is consistent with the mechanism proposed for DC resistance to NK killing.

### Pivotal role of HMGB1 in NK-dependent upregulation of c-IAP2 and c-FLIP in HIV-1-infected DCs

**Figure 6:**
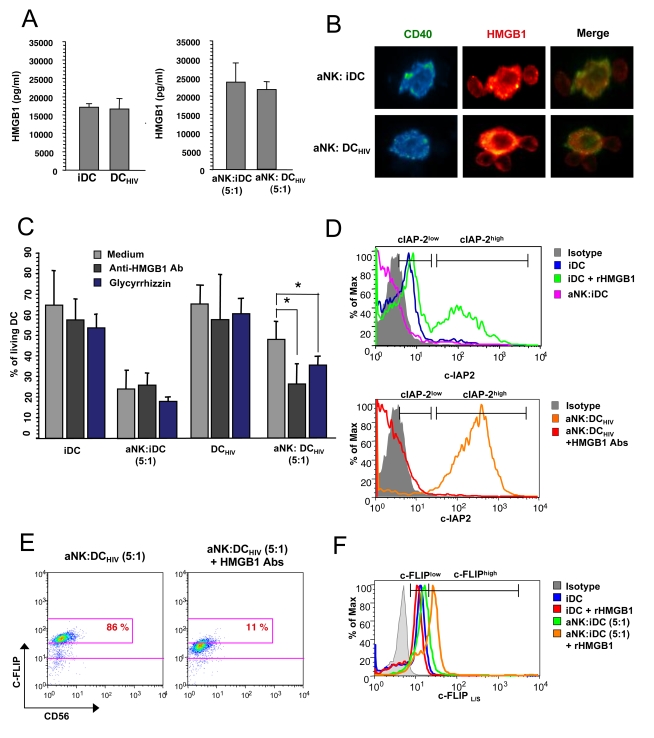
c-IAP-2 and c-FLIP upregulation in HIV-infected iDCs is triggered by HMGB1. (**A**) Detection of HMGB1, quantified by ELISA, in 24 h cell-free culture supernatants from iDCs, DC_HIV_ (left histogram) or from cocultures of aNK∶iDCs or aNK∶DC_HIV_ (right histogram). (**B**) Immunofluorescence analysis of intracellular HMGB1 expression in aNK-DC conjugates, iDCs being either uninfected (upper panel) or HIV-infected (lower panel). DCs were co-stained with CD40 specific antibodies. HMGB1 is detected both in aNK cells and DCs, whether infected or not. Pictures from one representative experiment out of three conducted with different primary cell preparations are shown. (**C**) iDCs or DC_HIV_ were cultured either alone or in the presence of aNK cells for 24 h. Blocking anti-HMGB1 antibodies (10 µg/ml), or glycyrrhizin (10 µg/ml), were added at coculture initiation. Cell death was quantified in DCs with the 7-AAD assay on gated CD56^neg^ cells. The mean ± sd of three independent experiments is shown. * p<0.05. (**D**) iDCs were cultured either alone, in the presence of recombinant HMGB1 (10 µg/ml), or with aNK cells. In some NK-DC cocultures, blocking anti-HMGB1 antibodies (10 µg/ml) were added. Intra-cellular expression of c-IAP2 in DCs was then analyzed by flow cytometry. Histograms are representative of one out of three independent experiments. (**E**) HIV-infected DCs were cocultured with aNK cells in the absence or presence of blocking anti-HMGB1 antibodies (10 µg/ml). Intra-cellular expression of c-FLIP in DCs was analyzed by flow cytometry. The percentage of c-FLIP^high^ DCs, gated as CD56^neg^ cells, is indicated. Dot plots are representative of one out of three independent experiments. (**F**) iDCs were cultured either alone, in the presence of rHMGB1, or cocultured with aNK cells in the absence or presence of rHMGB1 (10 µg/ml) during 24 h. Intra-cellular expression of c-FLIP in DCs (gated as CD56^neg^ cells) was analyzed by flow cytometry. Histograms are representative of one out of three independent experiments.

We recently reported that activated NK cells were able to induce the maturation of HIV-1-infected DCs, at NK-DC ratio of 1∶5, but it resulted in a dramatic increase in viral replication and proviral DNA expression in DCs [16]. This process was mainly triggered by HMGB1, released by both cell types, as a consequence of NK-DC cross-talk [7]. **Fig. 6A** confirms that both uninfected and HIV-1-infected iDC spontaneously released high levels of HMGB1 during a 24h culture. Their interaction with aNK cells at a 5∶1 ratio resulted in an enhancement of HMGB1 production in culture supernatant due to the additive effect of HMGB1 released by NK cells, as we previously showed [16]. Analysis of NK-DC conjugates by confocal microscopy confirmed the expression of HMGB1 both by NK cells and DCs, and whatever the infected status of DCs (**Fig. 6B**) [16]. The possible involvement of HMGB1 in the induction of the resistance state of DC_HIV_ to NK killing was tested in NK-DC co-cultures in the presence of either Glycyrrhizin, known to interact specifically with soluble HMGB1 [26], or anti-HMGB1 neutralizing antibodies. Both inhibitors, added at the initiation of the 24h aNK-iDC_HIV_ cocultures, restored the susceptibility of HIV-1-infected DCs to NK cytotoxicity, leading to a significant decrease in DC_HIV_ survival (**Fig. 6C**). These inhibitors had no impact on DC_HIV_ survival in the absence of NK cells. Simultaneous flow cytometry analysis of c-IAP2 expression revealed that anti-HMGB1 antibodies abrogated c-IAP2 up-regulation in DC_HIV_ (**Fig. 6D** lower panel) and, as a corollary, rHMGB1 was found to upregulate c-IAP2 in iDCs (**Fig. 6D** upper panel). Similar observations were made for c-FLIP. Anti-HMGB1 antibodies induced a strong decrease in c-FLIP expression in DC_HIV_ cocultured with aNK cells (**Fig. 6E**), while rHMGB1 increased c-FLIP expression in iDC when cocultured with aNK cells (**Fig. 6F**). Altogether, these observations argue for a pivotal role of HMGB1 in the acquired resistance of HIV-1-infected DCs to NK cytotoxicity, preventing TRAIL-induced apoptosis by upregulating two potent inhibitors, c-IAP2 and c-FLIP.

In a previous study, we reported that NK∶DC_HIV_ cognate interaction under conditions of DC maturation (ratio 1∶5) resulted in a strong increase in HIV-1 viral replication in DCs (detected at day 3 after infection), that was mediated by HMGB1 [16]. We confirmed these observations in the present study, showing that NK-DC_HIV_ cross-talk under conditions of protection of DC_HIV_ from TRAIL-induced apoptosis stimulates HIV-1 replication in DC_HIV_, measured either as p24 release in culture supernatant of following intracellular detection of p24 in DCs (data not shown). Thus, DC escape from NK cytotoxicity contributes to viral dissemination and persistence.

## Discussion

In opposition to the immune sentinel function of DCs to capture and present processed antigens from pathogens, HIV-1 hijacks DCs to promote viral dissemination. In a recent study, we reported that the cross-talk between NK cells and HIV-1-infected DCs, under conditions of DC maturation, resulted in a dramatic increase in viral replication and proviral DNA in DCs, and this process was mainly triggered by HMGB1, a DC maturation factor produced in NK-DC cocultures [16]. In the present study, we show for the first time that NK-DC cross-talk, under conditions of killing of iDC by NK cells, results in a strong resistance of HIV-1 infected DCs to NK cytotoxicity, preventing elimination of infected DCs and thus contributing to viral dissemination. HMGB1 was found to be a key factor in DC resistance to NK killing, inducing the upregulation of c-FLIP and c-IAP2 in infected DCs, thus preventing TRAIL-dependent NK-mediated cytotoxicity. Therefore, cognate interactions between NK cells and DCs, required for regulation of innate immunity [6], and found *in vivo* to be integral to the activation of effective antiviral immunity [22], become detrimental to the host in the context of HIV infection.

NK cells have the capacity to spontaneously kill tumor cell lines, in contrast they do not generally kill non transformed autologous cells. NK cells were recently shown to play a relevant role in the process of DC maturation, either by direct DC stimulation or through killing those DCs that did not properly acquire a mature phenotype (‘DC editing’) [27] [6] [28]. Killing of autologous immature DCs was reported to involve NK-p30 [17] and DNAM-1 [19] and to be confined to NK-cells expressing CD94-NKG2A inhibitory receptor [20]. While the NK receptors involved in NK-mediated DC killing have been, at least in part, identified, the molecular pathway whereby NK cells kill DCs is less understood. In the present study, we identified the cytotoxicity pathway used by human NK cells to kill autologous iDCs and found that the TRAIL/DR4 pathway was used rather than the perforin pathway. TRAIL is a member of the TNF ligand family that signals apoptosis via the death domain–containing receptors TRAIL-R1 (DR4) and TRAIL-R2 (DR5). It is primarily expressed as a type II membrane protein (mTRAIL) and is also secreted in a soluble form (sTRAIL) only by activated T and NK cells. In the present study, we found mTRAIL expression confined to CD56^bright^ cells, whereas perforin expression was restricted to CD56^dim^ cells and independent of NK-cell activation state (in agreement with previous reports [29]). Activation of NK cells triggered TRAIL release, and cognate interaction with iDCs induced DR4 expression on a fraction of them, which became susceptible to TRAIL-mediated apoptosis. iDCs by themselves were not able to produce TRAIL. Preventing TRAIL-DR4 interaction with specific blocking antibodies inhibited NK-mediated iDC killing, while the perforin-based cytotoxicity inhibitor concanamycin A had no effect. These data are consistent with a previous study showing that *in vivo* elimination of iDCs by murine NK cells is TRAIL-dependent [30]. Surface-bound TRAIL is one of the effector mechanisms of NK cells in suppression of tumor cell growth *in vivo* [31], and it selectively kills virus-infected cells while leaving uninfected cells intact [32]. We report herein for the first time that TRAIL is the major effector mechanism of iDC editing by human NK cells.

The contribution of NK-DC crosstalk to the control of viral infections is poorly documented. Andoniou *et al.* demonstrated, in an *in vivo* murine model of MCMV infection, that virus-infected DCs are capable of enhancing NK-cell cytotoxicity and NK cell-dependent clearance of the virus [22], arguing for a protective role of innate cell cross-talk. Nevertheless, HIV-1 seems to exploit this cross-talk to its own advantage. Indeed, abnormalities were reported in the *in vitro* interactions between NK cells and autologous DCs from viremic HIV-1-infected patients, including an impairment in NK-cell-mediated elimination of iDCs [24]. It has been attributed to defective NK-cell mediated killing associated to impaired expression and function of NKp30 and TRAIL by patients' NK cells [24]. In the present study, we addressed whether infected DCs had any role in the impairment of NK cells to kill them. We report for the first time that HIV-1 infection protects DCs from NK cell cytotoxicity through a mechanism involving the cognate interaction between aNK cells and DC_HIV_, which rescues DC_HIV_ from TRAIL-mediated killing by upregulating two potent inhibitors of apoptosis, c-FLIP and c-IAP2. Apoptosis is a tightly regulated cell process where intracellular proteins c-FLIP [33] and c-IAPs [34], [35] are major players, blocking the death receptor signaling pathway by preventing caspase-8 activation [36] at the death-inducing signaling complex (DISC) [37], and inhibiting the effector capsase-3, respectively. Thus, while c-IAP2 and c-FLIP were expressed by all iDCs, whether infected or not, cognate interaction between aNK cells and iDCs upregulated the expression of both inhibitors of apoptosis in HIV-1-infected DCs only, making these cells resistant to NK killing. From these observations we concluded that c-IAP2^bright^ and c-FLIP^bright^ DCs acquired a state of resistance to TRAIL-mediated NK cytotoxicity. We also discovered that DC_HIV_ rescuing process was mediated by the alarmin HMGB1, essential for the promotion of DC maturation and elicitation of an immune response [13], [38]. HMGB1, detected in the nucleus of sorted primary NK cells [16] and translocated to the cytoplasm following their activation [16], was also expressed in DCs, whether infected by HIV-1 or not. HMGB1 was also released by both cells in culture supernatants, at similar levels in iDCs or DC_HIV_ and in aNK-iDC or aNK-DC_HIV_ cocultures. In a recent study, it was proposed that HMGB1 secretion by NK cells is dependent upon IL-18 released by iDC at the synaptic cleft, inducing DC maturation and protection from lysis [7]. However, the mechanism involved in DC protection from NK lysis was unknown, and our study is the first to report a strong inducer effect of HMGB1 on c-FLIP and c-IAP2 expression as a survival mechanism. Our observations are compatible with a recent study reporting in human colon carcinomas a correlation between increased HMGB1 levels and enhanced amounts of c-IAP2 [39].

Since DCs are located in the mucosa [1] and thought to be among the first cells that encounter HIV-1 during sexual transmission [40] and disseminate the virus in lymphoid organs, it was important to determine the consequences of aNK-iDCs interaction on viral replication and persistence. Our previous study [16] and this one strongly argue for a deleterious role of NK cells, through HMGB1 release, on HIV-1 control. Indeed, under conditions of iDC maturation (NK∶DC ratio of 1∶5) [16] and iDC killing (NK∶DC ratio 5∶1) (not shown), aNK-DC_HIV_ interaction led in a few hours to a significant increase in the frequency of infected DCs and in the production of viral particles [16]. Furthermore, under conditions of DC killing, aNK-DC_HIV_ interaction led to the resistance of infected DCs to NK cytotoxicity. The pivotal role of HMGB1 in that context is puzzling. It was reported to activate replication of latent HIV-1 in infected monocytic cell lines [41] but also in primary infected DCs [16], but in some cases HMGB1 was found to inhibit viral production by macrophages, either by increasing the release of CCR5-interacting chemokines (RANTES, MIP-1α and MIP-1ß) [41] or by another mechanism to be determined [42]. Nevertheless, our report reveals that HMGB1 is essential for the survival of infected DCs, promoting the up-regulation of potent suppressors of apoptosis, c-FLIP and c-IAP2. Interestingly, the resistance of infected DCs to NK killing required productive HIV-1 infection, as the addition of AZT at the time of DC infection preserved their susceptibility to NK killing. Since specific inhibitors of HMGB1 also allowed productively infected DCs to be killed by NK cells, these observations indicate a conjugated effect of both HMGB1 and HIV on the acquired resistance of DC_HIV_ to TRAIL-induced apoptosis. Inhibition of the apoptotic response to TRAIL in many cancer cells involves NF-κB activation that upregulates, among anti-apoptotic molecules, c-FLIP and c-IAP2 [43] [44]. In our study, the involvement of NF-κB in TRAIL-induced resistance of DC_HIV_ is suggested by the recent demonstration of the ability of HMGB1 to increase c-IAP2 expression levels in carcinoma cells by enhancing NF-κB activity [39], and the requirement for HIV to promote its replication and prevent host-cell apoptosis by activating NF-κB [45]. This hypothesis is currently under investigation.

Thus, from our previous data [16], coupled with the results presented here, we conclude that certain bidirectional NK-DC interactions, that normally occur after an inflammatory insult, are altered after infection of iDCs with HIV-1, resulting in (i) the abnormal maturation of DC_HIV_ that are impaired in their ability to secrete IL-12 and IL-18 and to prime Th1 cells [16], (ii) increased expression of HIV-RNA and HIV-DNA in mature DC_HIV_ [16], (iii) deficient killing of infected DCs by NK cells due to HMGB1-dependent upregulation of the anti-apoptotic molecules c-FLIP and c-IAP2 in DC_HIV_ (the present study). The pivotal role of HMGB1 in NK-DC_HIV_ interaction was highlighted by the observation that it was responsible for promoting the protection of DC_HIV_ from NK-dependent cytotoxicity, and also involved in the triggering of HIV-1 replication in DC_HIV_ [16]. The role of HMGB1 in HIV disease is currently unknown, though plasma levels were reported to be elevated in HIV-1-infected patients compared to healthy donors, with the highest concentrations found in patients with clinical complications [46]. Moreover, circulating HMGB1 levels were found correlated with HIV-RNA plasma viral load (M-L Gougeon et al. unpublished data). Altogether these observations provide evidence for the crucial role of NK-DC cross-talk in promoting viral dissemination and maintaining viability of long-term reservoirs in DC population, they challenge the question of the *in vivo* involvement of HMGB1 in the triggering of viral replication and persistence of these reservoirs, and they suggest that c-FLIP or c-IAP2 molecules may represent therapeutic targets for the destruction of infected DCs.

## Materials and Methods

### Isolation and preparation of iDC and NK cells

Peripheral Blood Mononuclear Cells (PBMCs) were separated from the blood of healthy adult donors on a Ficoll-Hypaque density gradient. Blood was obtained through the EFS (Etablissement Français du Sang) in the setting of EFS-Institut Pasteur Convention. A written informed consent was obtained for each donor to use the cells for clinical research according to French laws. Our study was approved by IRB, external (EFS Board) as required by French law and internal (Biomedical Research Committee Board, Institut Pasteur) as required by Institut Pasteur.

We isolated CD14^+^ monocytes from PBMCs by positive selection using CD14-specific immunomagnetic beads (Miltenyi Biotech). To generate iDCs, purified CD14^+^ monocytes (purity >95%) were cultured for 5 days (10^6^ cells/ml) in RPMI 1640 medium supplemented with 2mM glutamine, 10% FCS, penicillin (100 U/ml) and streptomycin (100 µg/ml), in the presence of 10 ng/ml of recombinant human (rhu) GM-CSF and 10 ng/ml rhuIL- 4 (Peprotech), as described [47]. Culture medium was replaced every 2 days. CD56^+^ NK cells were isolated by negative selection from PBMCs using the «EasySep NK depletion Kit» (StemCell Technologies). NK cell fraction (CD3^−^CD56^+^) was more than 95% pure, as assessed by flow cytometry (FACScalibur, BD) using FITC-conjugated anti-CD3 and APC-conjugated anti-CD56 antibodies. Contamination with myeloid cells, assessed with FITC-conjugated anti-CD14 antibodies, was consistently less than 1%. Purified NK cells were cultured at 10^6^cells/ml for 48 hours either in the presence of suboptimal concentration of IL-2 (100 ng/ml) (Peprotech) to maintain their viability (referred as rNK), or were activated by a combination of PHA (10 µg/ml) (Sigma) and rhuIL-2 (10 µg/ml) (referred as aNK cells), before launching NK-DC coculture experiments. In experiments analyzing CD107a expression, NK cells were stimulated for 24 h with PMA (50 ng/ml) (Sigma) and ionomycin (300 ng/ml) (Sigma).

### Infection of iDCs with HIV-1

Virus stock was prepared by amplification of R5-HIV-1_BaL_ on Monocytes-derivMDM. Viral stock was then clarified by centrifugation prior to determination of HIV-1 p24 concentration. iDCs were plated in 96-well culture plates at 200,000 cells/well and incubated for 24 hours at 37°C in a 5% CO_2_ atmosphere with HIV-1 at 1 ng p24/ml. Cells were harvested, washed three times with media containing 10% FCS and, when indicated, rNK or aNK cells were added at a NK∶DC ratio of 5∶1. NK-DC cocultures lasted 24h, unless otherwise indicated, before analysis of DCs phenotype, viability, or quantification of viral production. In some experiments, HIV-1 infected iDCs were incubated alone or with aNK cells, in the presence of rhu-HMGB1 (1 µg/ml) (R&D Systems), blocking anti-HMGB1 Abs (10 µg/ml) (Abcam), Glycyrrhizin (10 µg/ml) (Sigma-Aldrich) or Bisindolylmalmeimide III (25µM) (Alexis Biochemicals). HIV-1 concentration in culture supernatant was determined with the p24 ELISA kit (Ingen). The frequency of HIV-1-infected cells was determined by flow cytometry to detect intracellular p24 molecule. Cells were surface stained with antibodies specific for CD40 (BD) to target DC and intracellular stained with p24-specific antibodies (Beckman Coulter). Stained cells were immediately acquired on a FACScalibur and analyzed with FlowJo software.

### Phenotypic analyses by flow cytometry

Surface staining of DCs was performed with anti-CD40, -HLA-DR, -CD83, -CD86, -HLA-E or -DC-SIGN (BD Biosciences), -DR4 or -DR5 (e-Bioscience) mAbs conjugated to FITC, PE or APC. Surface staining of NK cells was performed with anti-mTRAIL (e-Bioscience), -CD56 or -CD107a (BD) mAbs conjugated to PE or APC. Cells were stained for 30 minutes at 4°C, washed twice in PBS/BSA/NaN_3_ (0.5% BSA, 0.01% NaN_3_), fixed with 1% PFA, acquired on a FACScalibur and analyzed with FlowJo software. Populations of interest were selected by gating according to FSC/SSC parameters, then targeting DCs as CD56^−^ cells and NK cells as CD56^+^ cells. For intracellular staining, cells were fixed with 4% PFA, permeabilized using 0.5% BSA, 0.01% NaN_3_, 0.5% Saponin buffer, stained for 20 minutes at room temperature with FITC-labeled anti-perforin mAbs (BD), PE-conjugated anti-p24 mAbs (Beckman Coulter), PE-conjugated anti-active caspase-3 Abs (BD), unconjugated rabbit anti-human c-IAP2 polyclonal antibody (clone H-85, Santa Cruz Biotechnology) or c-FLIP antibodies (Santa Cruz biotechnology). For indirect staining, donkey anti-rabbit PE-conjugated secondary antibodies (Abcam) were used. Samples were acquired on a FACSCalibur and analyzed with FlowJo software.

### Confocal microscopy

Cells were stained with anti-CD40 mAbs (BD), anti-HMGB1 (Abcam) or anti-c-IAP2 unconjugated rabbit polyclonal antibodies (clone H-85, Santa Cruz Biotechnology). Goat anti-rabbit Cy5-conjugated secondary antibodies (Abcam) were used. Cells were washed in PBS, fixed on poly-L-lysine coated slides (Kindler, Freiburg, Germany), and mounted in an anti-fade DAPI Fluoromount-G (Southern Biotech). Slides were observed using a Zeiss Axiovert 200M Perkin-Elmer Spinning Disk equipped with a Hamamatsu ORCA II ER camera.

### NK-DC coculture experiments and apoptosis measurement

rNK or aNK cells were cocultured during 24h with uninfected or HIV-infected iDCs at different NK∶DC ratios: 1∶5 (2×10^5^ NK+10^6^ DCs/ml), 1∶1 (2×10^5^ NK+2×10^5^ DC/ml) and 5∶1 (1×10^6^ NK+2×10^5^ DC). In some experiments, mature DCs (DC0) were used as controls. DC maturation was induced by 48h stimulation of iDCs (10^6^/ml) with 10 µg/ml LPS (E. Coli serotype 026-B6, Sigma-Aldrich). DC survival was determined with the 7-AAD assay, as described previously [25]. Briefly, cultured cells were stained with 20 µg/mL nuclear dye 7-amino-actinomycin D (7-AAD; Sigma-Aldrich) for 30 minutes at 4°C, and co-stained with CD56-specific antibody (BD). Surviving DCs were identified as CD56^−^ 7-AAD^−^ FSC^high^ cells. When phenotypic characterization of DCs was performed in NK-DC cocultures, NK cells were always excluded from the FACS analysis through their staining with CD56-specific antibodies.

In some experiments, iDCs were incubated in the presence of rhuTRAIL (1–1000 ng/ml), (Alexis Biochemicals) or neutralizing antibodies i.e. anti-TRAIL (1 µg/ml), anti-DR4 (250 ng/ml) (R&D) or anti-Fas ligand (250 ng/ml). Concanamycin A (Alexis Biochemicals) was used at 100 nM for blocking perforin-mediated cytotoxicity in cocultures. Cell free culture supernatants were tested for soluble TRAIL and HMGB1 levels using ELISA kit from Diaclone, and Shino-test ELISA kit (IBL), respectively.

### Silencing experiments

siRNAs against c-IAP2, c-FLIP and control FITC-labeled siRNA were purchased from SantaCruz technologies. iDCs or HIV-infected DCs were seeded at 3×10^4^ cells/ml in 96-well plates. Gene-specific siRNAs and the control siRNA (0.1–50nM) were added to media containing *Polymag magnetofection* beads (OZ Biosciences) and incubated 20 minutes at room temperature. Cells were transfected either with *Polymag* beads alone, control FITC-labeled siRNA, c-IAP2 siRNA or with c-FLIP siRNA in a strong magnetic field (Magnetic plate, OZ Biosciences) for 20 minutes at 37°C, 5% CO_2_. iDCs were kept in culture in complete medium for 24h before measuring cell death.

### Microarray experiments

Total RNA was isolated from cocultures of aNK∶iDC or aNK∶DC_HIV_, and microarray experiments were performed by Miltenyi Biotech. Cells were centrifuged at 300g and resuspended in 1 ml Lysis/Binding Buffer. Lysate was sheared through a 21G needle and cleared using a LysateClear Column. Fifty microliter Oligo(dT) MicroBeads were added to the lysate and mRNA fixed and purified on a micro column. mRNA was transcribed to labeled cDNA using the thermo MACS Separator (Miltenyi Biotech) in a 60 min, 42°C in-column incubation with cDNA labeling mix and 1 µl Cy3-dCTP or Cy5-dCTP (1 mM, GE Healthcare). Labeled cDNA/mRNA hybrids were washed and RNase H digested in a 5 min, 42°C in-column incubation. cDNA was eluted with 50 µl cDNA Elution Buffer. PIQOR Microarray Immunology, human, sense (Miltenyi Biotech) hybridization was performed according to the manufacturer's instructions using an automized hybridization machine (a-Hyb, Miltenyi Biotech). Microarrays were blow-dried and scanned with the ScanArray Lite (GSI Lumonics) and the Agilent DNA-Microarray Scanner. Signal processing and quantification was conducted with ImaGene software version 5.0 (BioDiscovery). Local background was subtracted from the signal to obtain the net signal intensity and the ratio of Cy5/Cy3. Subsequently, the mean of the ratios of four corresponding spots representing the same cDNA was computed. Cluster analysis of pro-apoptotic and anti-apoptotic gene expression was performed by Miltenyi Bioinformatic services. Pearson correlation coefficient analysis was performed with the unfiltered ratio 2bkg dataset logarithmized to the basis of two. One and two dimensional average linkage hierarchical clustering using Euclidean distance as well as statistical analysis of microarrays (SAM)4 was performed with TIGR MeV version TM4 setting the percentage cutoff filter to 30%.

### Live videomicroscopy

aNK cells were added to iDCs at aNK∶iDC ratio of 5∶1. Cells were centrifuged and the pellet incubated for 15 minutes at 37°C in a 5% CO_2_ atmosphere to favor cell contacts. The pellet was then reconstituted in complete culture medium and cells were seeded at a concentration of 250 000 cells/ ml in a 35 mm microdish (Ibidi, France). The culture was observed in bright field using an inverted microscope Zeiss Axiovert 200M. Cells were maintained in a 37°C, 5% CO_2_ chamber while videos were recorded.

### Stastistical analyses

Statistical analyses were made with the non-parametric Mann-Whitney test. The *P* value of significant differences is reported. Plotted data represent mean ± standard deviation (s.d.).

## Supporting Information

Figure S1 CFSE-stained aNK cells induce apoptosis of immature DCs in aNK-DC cocultures but do not die. (A) CD56^+^ NK cells sorted from PBMC were activated with PHA+IL-2 (aNK) and cultured for 24 h either alone or in the presence of iDCs generated from purified CD14^+^ monocytes in the presence of IL-4 and GM-CSF (NK∶DC ratio 5∶1). The survival of CD3^neg^CD56^+^ cells was determined by flow cytometry with the 7-AAD assay. Apoptotic NK cells were identified as CD3^neg^ CD56^+^ 7-AAD^+^ cells. aNK cells cocultured with iDCs are not induced to die. Dot plots are representative of three independent experiments. (B) aNK cells and iDCs were cultured either separately or co-cultured at 5∶1 aNK∶DC ratio during 24 h. aNK cells were stained with CFSE prior to their culture in order to further identify them by FACS analysis. The death of DCs, gated as CFSE^neg^ cells, was determined by flow cytometry combining FSC parameter and 7-AAD staining. Three populations could be identified: 7-AAD^+^ cells corresponding to apoptotic cells, 7-AAD^neg^ FSC^low^ corresponding to apoptotic bodies, and 7-AAD^neg^ FSC^high^ corresponding to living cells. Comparison of aNK∶iDC panel with iDCs panel shows an increase in apoptotic cells and apoptotic bodies in DCs (CFSE^neg^) when cocultured with aNK cells. These dot plots are representative of at least 3 experiments.
(3.59 MB TIF)
