# Supplementary figures and images for: Escape of HIV-1-Infected Dendritic Cells from TRAIL-Mediated NK Cell Cytotoxicity during NK-DC Cross-Talk—A Pivotal Role of HMGB1
Source: PLoS Pathog. 2010 Apr 15;6(4):e1000862. doi: 10.1371/journal.ppat.1000862 (PMC2855334; doi:10.1371/journal.ppat.1000862)

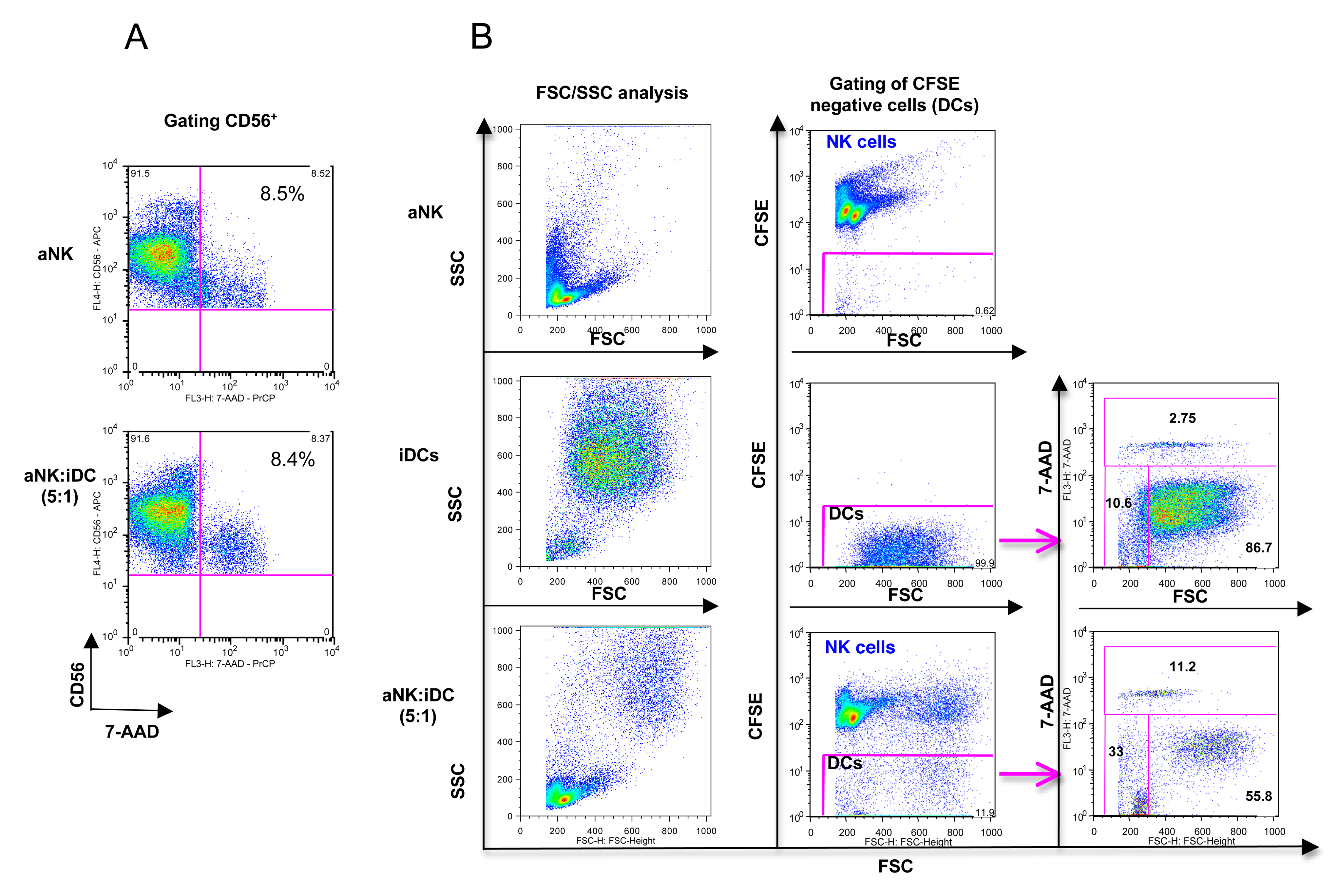

Supplement: Figure S1 — CFSE-stained aNK cells induce apoptosis of immature DCs in aNK-DC cocultures but do not die. (A) CD56+ NK cells sorted from PBMC were activated with PHA+IL-2 (aNK) and cultured for 24 h either alone or in the presence of iDCs generated from purified CD14+ monocytes in the presence of IL-4 and GM-CSF (NK∶DC ratio 5∶1). The survival of CD3negCD56+ cells was determined by flow cytometry with the 7-AAD assay. Apoptotic NK cells were identified as CD3neg CD56+ 7-AAD+ cells. aNK cells cocultured with iDCs are not induced to die. Dot plots are representative of three independent experiments. (B) aNK cells and iDCs were cultured either separately or co-cultured at 5∶1 aNK∶DC ratio during 24 h. aNK cells were stained with CFSE prior to their culture in order to further identify them by FACS analysis. The death of DCs, gated as CFSEneg cells, was determined by flow cytometry combining FSC parameter and 7-AAD staining. Three populations could be identified: 7-AAD+ cells corresponding to apoptotic cells, 7-AADneg FSClow corresponding to apoptotic bodies, and 7-AADneg FSChigh corresponding to living cells. Comparison of aNK∶iDC panel with iDCs panel shows an increase in apoptotic cells and apoptotic bodies in DCs (CFSEneg) when cocultured with aNK cells. These dot plots are representative of at least 3 experiments. (3.59 MB TIF) [file ppat.1000862.s001.tif]
